# Night shift work is associated with an increased risk of asthma

**DOI:** 10.1136/thoraxjnl-2020-215218

**Published:** 2020-11-16

**Authors:** Robert J Maidstone, James Turner, Celine Vetter, Hassan S Dashti, Richa Saxena, Frank A J L Scheer, Steven A Shea, Simon D Kyle, Deborah A Lawlor, Andrew S I Loudon, John F Blaikley, Martin K Rutter, David W Ray, Hannah Jane Durrington

**Affiliations:** 1 Division of Informatics, Imaging & Data Sciences, School of Biological Sciences, Faculty of Biology, Medicine and Health, University of Manchester, Manchester, UK; 2 Oxford Centre for Diabetes, Endocrinology and Metabolism, University of Oxford, Oxford, UK; 3 Medical School, University of Manchester, Manchester, UK; 4 Circadian and Sleep Epidemiology Laboratory, Department of Integrative Physiology, University of Colorado at Boulder, Boulder, Colorado, USA; 5 Program in Medical and Population Genetics, Broad Institute, Cambridge, Massachusetts, USA; 6 Center for Genomic Medicine, Massachusetts General Hospital, Boston, Massachusetts, USA; 7 Department of Anesthesia, Critical Care and Pain Medicine, Massachusetts General Hospital and Harvard Medical School, Boston, Massachusetts, USA; 8 Broad Institute of MIT and Harvard, Cambridge, Massachusetts, USA; 9 Medical Chronobiology Program, Division of Sleep and Circadian Disorders, Brigham and Women’s Hospital, Boston, Massachusetts, USA; 10 Division of Sleep Medicine, Harvard Medical School, Boston, Massachusetts, USA; 11 Oregon Institute of Occupational Health Sciences, Oregon Health & Science University, Portland, Oregon, USA; 12 Sleep and Circadian Neuroscience Institute, Nuffield Department of Clinical Neurosciences, University of Oxford, Oxford, UK; 13 MRC Integrative Epidemiology Unit, University of Bristol, Bristol, UK; 14 Population Health Sciences, Bristol Medical School, University of Bristol, Bristol, UK; 15 Division of Diabetes, Endocrinology & Gastroenterology, School of Medical Sciences, Faculty of Biology, Medicine and Health, University of Manchester, Manchester, UK; 16 Division of Infection, Immunity and Respiratory Medicine, School of Biological Sciences, Faculty of Biology, Medicine and Health, University of Manchester, Manchester, UK; 17 Wythenshawe Hospital, Manchester University NHS Foundation Trust, Manchester, UK; 18 Manchester Diabetes Centre, Central Manchester University Hospitals NHS Foundation Trust, Manchester Academic Health Science Centre, Manchester, UK; 19 NIHR Oxford Biomedical Research Centre, John Radcliffe Hospital, Oxford, UK, University of Oxford, Oxford, UK

**Keywords:** asthma epidemiology, asthma

## Abstract

**Introduction:**

Shift work causes misalignment between internal circadian time and the external light/dark cycle and is associated with metabolic disorders and cancer. Approximately 20% of the working population in industrialised countries work permanent or rotating night shifts, exposing this large population to the risk of circadian misalignment-driven disease. Analysis of the impact of shift work on chronic inflammatory diseases is lacking. We investigated the association between shift work and asthma.

**Methods:**

We describe the cross-sectional relationship between shift work and prevalent asthma in >280000 UK Biobank participants, making adjustments for major confounding factors (smoking history, ethnicity, socioeconomic status, physical activity, body mass index). We also investigated chronotype.

**Results:**

Compared with day workers, ‘permanent’ night shift workers had a higher likelihood of moderate-severe asthma (OR 1.36 (95% CI 1.03 to 1.8)) and all asthma (OR 1.23 (95% CI 1.03 to 1.46)). Individuals doing any type of shift work had higher adjusted odds of wheeze/whistling in the chest. Shift workers who never or rarely worked on nights and people working permanent nights had a higher adjusted likelihood of having reduced lung function (FEV_1_ <80% predicted). We found an increase in the risk of moderate-severe asthma in morning chronotypes working irregular shifts, including nights (OR 1.55 (95% CI 1.06 to 2.27)).

**Conclusions:**

The public health implications of these findings are far-reaching due to the high prevalence and co-occurrence of both asthma and shift work. Future longitudinal follow-up studies are needed to determine if modifying shift work schedules to take into account chronotype might present a public health measure to reduce the risk of developing inflammatory diseases such as asthma.

**Key messages**

**What is the key question?**

- Shift workers are at increased risk of cardiometabolic disorders and cancers. The mechanism is thought to involve circadian misalignment. In this study, using data from >280 000 individuals in the UK Biobank, we asked whether shift workers were at increased risk of asthma.

**What is the bottom line?**

- Shift workers, especially those working permanent night shifts, had an increased risk of asthma (especially moderate-severe asthma).

**Why read on?**

- The public health implications of our findings are potentially far-reaching, since both shift work and asthma are common in the industrialised world. Future longitudinal follow-up studies might determine whether an individual is suited to night shift work based on their chronotype. Currently there are no guidelines for the management of asthma in shift workers. We hope that our study might fuel future discussion in this area.

## Introduction

Most human biological processes are regulated by an internal circadian timing system to optimally prepare physiological functions for anticipated daily environmental and behavioural cycles. Cyclical light/dark environmental cues, mealtimes and physical activity can serve as 'Zeitgebers' for circadian timing. The development of artificial light has allowed extension of the active period of humans into the night, and through the night for night shift workers. This imbalance between our internal clock and the environment results in circadian misalignment.^1^ Shift work, a notable example of circadian misalignment, is invariably associated with sleep disruption and with increased risk of prevalent chronic diseases including metabolic and cardiovascular diseases^2 3^ and cancer.^4^ There is evidence of causal relationships between circadian misalignment and the development of metabolic and cardiovascular diseases.^5 6^

Approximately 20% of the working population in industrialised countries work permanent or rotating night shifts,^7^ exposing this large population to the risk of circadian misalignment-driven disease, making this is an important area of investigation. Analysis of the impact of shift work on chronic inflammatory diseases is lacking.

Asthma is a very common chronic inflammatory disease of the airways, affecting 339 million people worldwide^8^ and costing the UK public sector £1.1 billion.^9^ Intriguingly, asthma displays marked time of day variations in symptoms (wheeze and whistling),^10^ airway calibre^11^ and in underpinning inflammatory pathways.^12^ The physiological diurnal variation in airway calibre is under direct circadian control, independent of external environmental cues such as light/dark and fasting/feeding.^13^ In asthma it appears that the physiological diurnal variation in airway calibre is amplified, suggesting coupling between the internal body clock and pathogenic processes. This raises the possibility that misalignment between the internal body clock and the environment, such as that induced by night shift work, would impact on asthma risk. Indeed, a correlation between shift work and work-related asthma was found in a study of 544 individuals working in a cabling manufacturing plant^14^; however, small numbers and confounding limit generalisability. We therefore investigated the association between shift work and asthma in a much larger dataset from the UK Biobank^15^ in which we could also adjust for numerous major confounding factors.

We hypothesised that, when compared with day workers, shift work—especially involving nights—would be associated with a higher prevalence of asthma.

The primary outcome was risk of asthma in shift workers. Secondary outcome measures included risk of symptoms of asthma and lung function in shift workers, as well as assessment of the effects of lifetime duration of night shift work and night shift frequency on the risk of asthma.

We also investigated whether chronotype is associated with the risk of asthma in shift workers. Chronotype is the phenotypic expression of the internal circadian timing system and can affect how an individual adapts to shift work; earlier chronotypes experience shortened sleep duration and increased sleep disturbance during night shifts, whereas late chronotypes show similar disruption when working early shifts.^16^ Matching shift work patterns to chronotype can improve sleep quality and well-being.^17^

Lastly, we investigated the intersection between the genetic risk of asthma and shift work exposure. Asthma risk was captured using a genetic risk score (GRS), the sum of genetic variants with weighted effect sizes.^18^ If the asthma GRS affects the health impact of shift work exposure, this may provide an employment screening opportunity in the future.

## Methods

The UK Biobank recruited 502 540 participants (5% of those invited) aged 40–69 years who were registered with the National Health Service (NHS) and lived within reasonable travelling distance of 22 assessment centres across the UK between 2007 and 2010.^19^ At the baseline visit, participants completed questionnaires on lifestyle, medical history, occupation and work hours. Trained health professionals asked further details about medical conditons, health status and medications. The selection of participants analysed in all comparisons are shown in a STROBE diagram (online supplemental figure 1) and further information can be found in the online supplemental methods.

10.1136/thoraxjnl-2020-215218.supp1 Supplementary data

10.1136/thoraxjnl-2020-215218.supp2 Supplementary data

Analysis of shift work was restricted to participants in paid employment or who were self-employed at baseline (n=286 825, age range 37–72 years)^2^; we did not exclude any individuals based on other diagnoses. The demographics of this group are shown in table 1. Of these, 83% were day workers and 17% worked shifts, of which 51% included night shifts. Compared with day workers, shift workers were more likely to be male, lived in more deprived neighbourhoods (Townsend area deprivation Index), more likely to live in an urban area and more likely to be smokers. Shift workers drank less alcohol, reported shorter sleep duration and longer weekly working hours. Night shift workers were more likely evening chronotypes compared with those working days. Compared with day workers, shift workers were more likely to have a diagnosis of gastro-oesophageal reflux, chronic obstructive pulmonary disease (COPD)/emphysema, higher cholesterol and hypertension. Shift workers were more likely of non-European ancestry and to be in jobs linked to occupational asthma or to jobs that require a medical examination. There were differences between the proportion of workers in each employment category across the work schedules. Night shift workers were more likely to work in the service occupations or as process, plant and machine operatives. In contrast, day workers tended to be in administrative roles and in professional occupations (online supplemental table 1).

**Table 1:** Clinical characteristics by current shift work exposure (n=286 825)

	Current work schedule			
	Day workers	Shift work, but never or rarely night shifts	Irregular shift work including nights	Permanent night shift work
N	236 897	24 560	18 226	7142
Age, years	52.90 (7.13)	52.48 (7.08)	51.08 (6.87)	51.45 (6.91)
Sex (% male)	46.58	47.51	62.43	61.43
BMI, kg/m^2^	27.09 (4.65)	27.79 (4.99)	28.21 (4.91)	28.51 (4.88)
Smoker, %				
Never	58.10	53.66	52.82	51.99
Previous	31.91	32.11	30.52	30.03
Current	9.75	13.88	16.19	17.67
Smoking, pack-years	20.07 (16.07)	22.92 (17.49)	24.31 (17.77)	25.70 (18.38)
Daily alcohol intake, %	20.48	16.89	15.98	10.21
Sleep duration (hours)	7.05 (1.03)	6.95 (1.22)	6.85 (1.30)	6.67 (1.52)
Morning chronotype, %	23.33	25.49	22.85	19.24
Evening chronotype, %	8.02	7.87	9.83	16.90
Ethnicity (%)				
White British	88.47	83.30	79.87	80.99
White other	6.45	7.07	7.03	6.01
Mixed	0.65	0.90	0.97	0.87
Asian	1.72	3.58	3.84	3.39
Black	1.40	2.69	4.93	5.47
Chinese	0.34	0.48	0.46	0.67
Other	0.09	0.13	0.10	0.14
Weekly work hours	34.24 (13.19)	34.97 (13.21)	39.29 (14.55)	39.59 (13.73)
Job asthma risk (%)	7.59	7.18	8.11	7.74
Job medical required (%)	2.27	2.52	4.14	3.68
Single occupancy (%)	15.64	18.78	18.71	18.42
Urban area (%)	85.98	89.59	89.33	90.97
Townsend Index	−2.24 (−3.70 to 0.19)	−1.31 (−3.18 to 1.61)	−1.24 (−3.17 to 1.82)	−1.04 (−3.02 to 2.07)
Maternal smoking (%)	26.59	28.88	29.23	30.75
Breastfed as baby (%)	56.12	54.27	54.16	51.51
Birth weight (kg)	3.33 (0.63)	3.31 (0.68)	3.35 (0.67)	3.31 (0.71)
Hypertension (%)	19.75	21.58	21.64	22.81
High cholesterol (%)	7.88	8.55	8.54	9.27
Sleep apnoea (%)	0.28	0.30	0.42	0.27
COPD/ emphysema/chronic bronchitis (%)	0.81	1.26	1.27	1.23
Bronchiectasis (%)	0.14	0.12	0.03	0.14
Interstitial lung disease (%)	0.02	0.01	0.03	0.01
Other respiratory problems (%)	0.12	0.17	0.16	0.07
Gastro-oesophageal reflux (%)	3.19	3.65	3.84	4.16

Data are mean (SD), median (IQR) or percentages. Positive values of the Townsend Index indicate high material deprivation, negative values indicate relative affluence. The diagnosis of conditions (hypertension, high cholesterol, sleep apnoea, COPD/emphysema/chronic bronchitis, bronchiectasis, interstitial lung disease, other respiratory problems and gastro-oesophageal reflux) came from participants self-reporting a doctor diagnosis.

BMI, body mass index; COPD, chronic obstructive pulmonary disease.

### Cases of asthma

Cases of asthma were defined by including all participants with self-reported doctor-diagnosed asthma at baseline who were also receiving any asthma medication.^20^ Using these criteria, we identified 14 238 (5.3%) cases, of which 4783 (1.9%) had moderate-severe asthma (defined as having doctor-diagnosed asthma at baseline and currently taking medication in accordance with steps 3–5 of the British Thoracic Society guidance for the treatment of asthma).^20^ We excluded from our analyses participants with doctor-diagnosed asthma who did not report taking asthma medication as well as those participants reporting taking asthma medication who did not have doctor-diagnosed asthma (n=20 151). For analysis of moderate-severe asthma, we further excluded those not on medication for moderate-severe asthma (listed in the Methods section, n=9455). Initially, we focused on those with moderate-severe asthma, since these individuals were more likely to have active asthma requiring regular disease-modifying treatment, so reducing the risk of misdiagnosis.

### Statistical analysis

We fitted a multivariate logistic regression model to the data and used this to estimate adjusted ORs and 95% asymptotic CIs on those ORs.

In model 1 we initially adjust for participant age and sex. We extend this in model 2 to additionally include BMI, ethnicity, chronotype, Townsend Deprevation Index (TDI), days exercised, smoker status (current, previous or never) and pack-years smoked, alcohol status (current, previous or never) and alcohol weekly intake, length of working week and whether current job is considered to have an occupational asthma risk or requires a medical examination prior to hiring. These covariates were chosen by consideration of participant characteristics (table 1). Model 3 also included sleep duration in addition to covariates in model 2.

## Results

**Figure 1:**
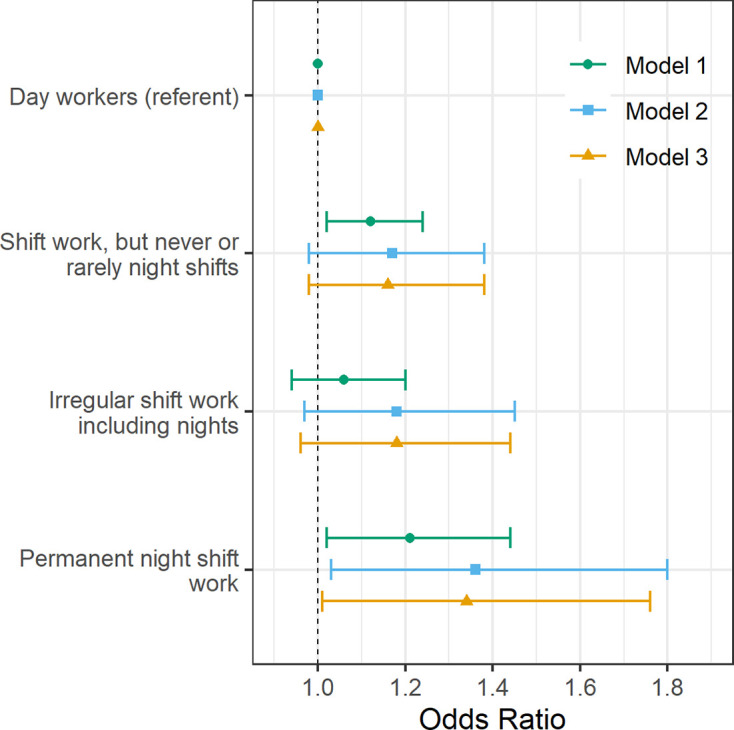
Adjusted odds of moderate-severe asthma by current shift work exposure (n=257 219). Forest plot of adjusted ORs with corresponding 95% asymptotic CIs for moderate-severe asthma stratified by current work pattern. Three multivariate logistic regression models were fitted to the data: Model 1 (green circle): age- and sex-adjusted. Model 2 (blue square) covariates: age, sex, smoking status, smoking pack-years, alcohol status, daily alcohol intake, ethnicity, Townsend Deprivation Index, days exercised (walked, moderate and vigorous), body mass index, chronotype, length of working week, job asthma risk and job medical required. Model 3 (yellow triangle): model 2 covariates plus sleep duration.

In an age- and sex-adjusted model, there were higher odds of having moderate-severe asthma in shift workers who never or rarely undertook night shifts (OR 1.12 (95% CI 1.02 to 1.24) and in those on permanent night shifts (OR 1.21 (95% CI 1.02 to 1.44)) when compared with day workers (figure 1). After further adjusting for smoking status and pack years, alcohol status and intake, ethnicity, social deprivation, physical activity, BMI, chronotype, length of working week, job asthma risk and job medical examination required (Model 2), associations attenuated in shift workers who never or rarely undertook night shifts (OR 1.17 (95% CI 0.98 to 1.38)) and slightly increased in permanent night shift workers (OR 1.36 (95% CI 1.03 to 1.8)). Further adjustment for sleep duration had no additional effects on the estimates (Model 3).

A similar pattern of higher odds of asthma was seen when all cases of asthma were considered (see online supplemental table 2). In an age- and sex-adjusted model, we observed higher odds of asthma in shift workers who never or rarely worked night shifts when compared with day workers (OR 1.08 (95% CI 1.02 to 1.15)). However, this association attenuated to the null with covariate adjustment (Model 2). The odds of asthma in shift workers working permanent nights were higher in covariate-adjusted models (Model 2: OR 1.23 (95% CI 1.03 to 1.46); Model 3: OR 1.20 (95% CI 1.01 to 1.43)) than in the age- and sex-adjusted model.

### Symptoms of asthma

**Figure 2:**
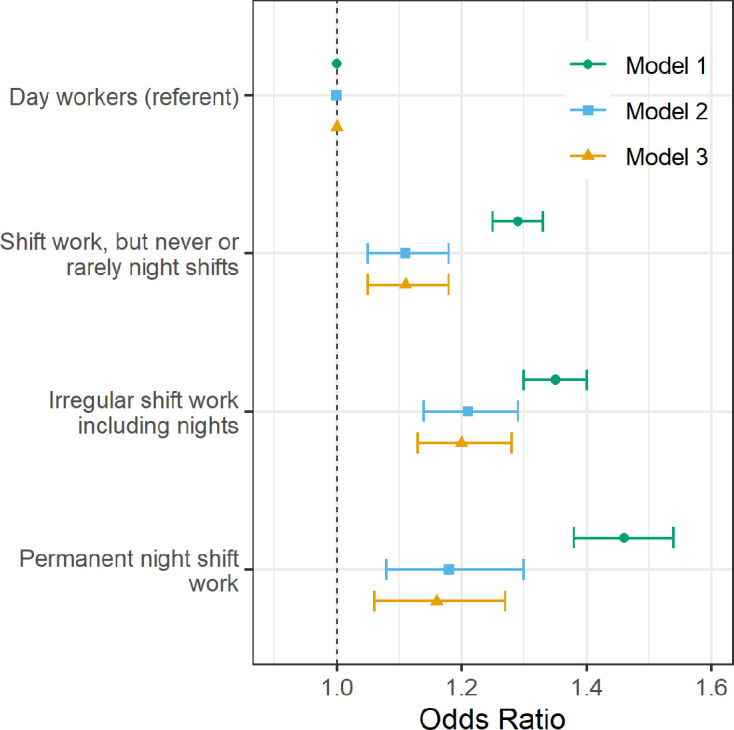
Adjusted odds of experiencing wheeze or whistling in the chest in the last year by current shift work exposure (n=280 998). Forest plot of adjusted ORs with corresponding 95% asymptotic CIs for experiencing wheeze or whistling in the chest in the last year stratified by current work pattern. Three multivariate logistic regression models were fitted to the data: Model 1 (green circle): age- and sex-adjusted. Model 2 (blue square) covariates: age, sex, smoking status, smoking pack-years, alcohol status, daily alcohol intake, ethnicity, Townsend Deprivation Index, days exercised (walked, moderate and vigorous), body mass index, chronotype, length of working week, job asthma risk and job medical required. Model 3 (yellow triangle): Model 2 covariates plus sleep duration.

We then analysed the association between shift work and the experience of wheeze or whistling in the chest in the previous year (n=280 998). When compared with day workers, the age- and sex-adjusted model revealed higher odds for these symptoms in association with all three types of shift work (shift work but never or rarely night shifts, irregular night shifts and permanent nights; figure 2. These associations with wheeze or whistling were attenuated but remained statistically significant for all types of shift work in Models 2 and 3 (eg, Model 2: shift work, but never or rarely night shifts: OR 1.11 (95% CI 1.05 to 1.18); irregular shift work including nights: 1.21 (95% CI 1.14 to 1.29); and permanent night shift work: 1.18 (95% CI 1.08 to 1.30)).

### Reduced lung function

We also examined the association between shift work status and reduced lung function assessed as the proportion of participants with a forced expiratory volume in 1 s (FEV_1_) that was <80% of the predicted value based on height and age (n=89 157). In age- and sex-adjusted models there were higher odds of participants having an FEV_1_ <80% predicted in all shift work groups compared with day workers (table 2). After multivariable adjustment, these associations attenuated towards the null with higher odds remaining for shift workers who never or rarely worked night shifts and for those working permanent nights (eg, Model 2: shift work, but never or rarely night shifts: OR 1.19 (95% CI 1.08 to 1.32); permanent night shift work: OR 1.20 (95% CI 1.03 to 1.41) compared with day workers).

**Table 2:** Adjusted odds (95% CI) of having a critical FEV_1_ predicted percentage (<80%) by current shift work exposure (n=89 157)

	Current work schedule			
	Day workers	Shift work, but never or rarely night shifts	Irregular shift work including nights	Permanent night shift work
Total cases (% of total sample size)	9381 (12.73%)	1183 (15.84%)	849 (15.07%)	397 (16.99%)
Total sample size	73 719	7469	5632	2337
Model 1: Age- and sex-adjusted OR (95% CI)	1 (referent)	1.31 (1.23 to 1.40)	1.27 (1.18 to 1.37)	1.44 (1.29 to 1.61)
Model 2: Multivariable adjusted OR (95% CI)	1 (referent)	1.19 (1.08 to 1.32)	1.08 (0.96 to 1.21)	1.20 (1.03 to 1.41)
Model 3: Model 2 covariates+sleep duration OR (95% CI)	1 (referent)	1.19 (1.08 to 1.32)	1.08 (0.97 to 1.21)	1.21 (1.04 to 1.42)

The predicted FEV_1_ was estimated based on height and age. Model 2 covariates: age, sex, smoking status, smoking pack-years, alcohol status, daily alcohol intake, ethnicity, Townsend Deprivation Index, days exercised (walked, moderate and vigorous), body mass index, chronotype, length of working week, job asthma risk and job medical required. Model 3 data are adjusted for Model 2 covariates plus sleep duration.

FEV_1_, forced expiratory volume in 1 s.

### Lifetime duration of night shift work

**Figure 3:**
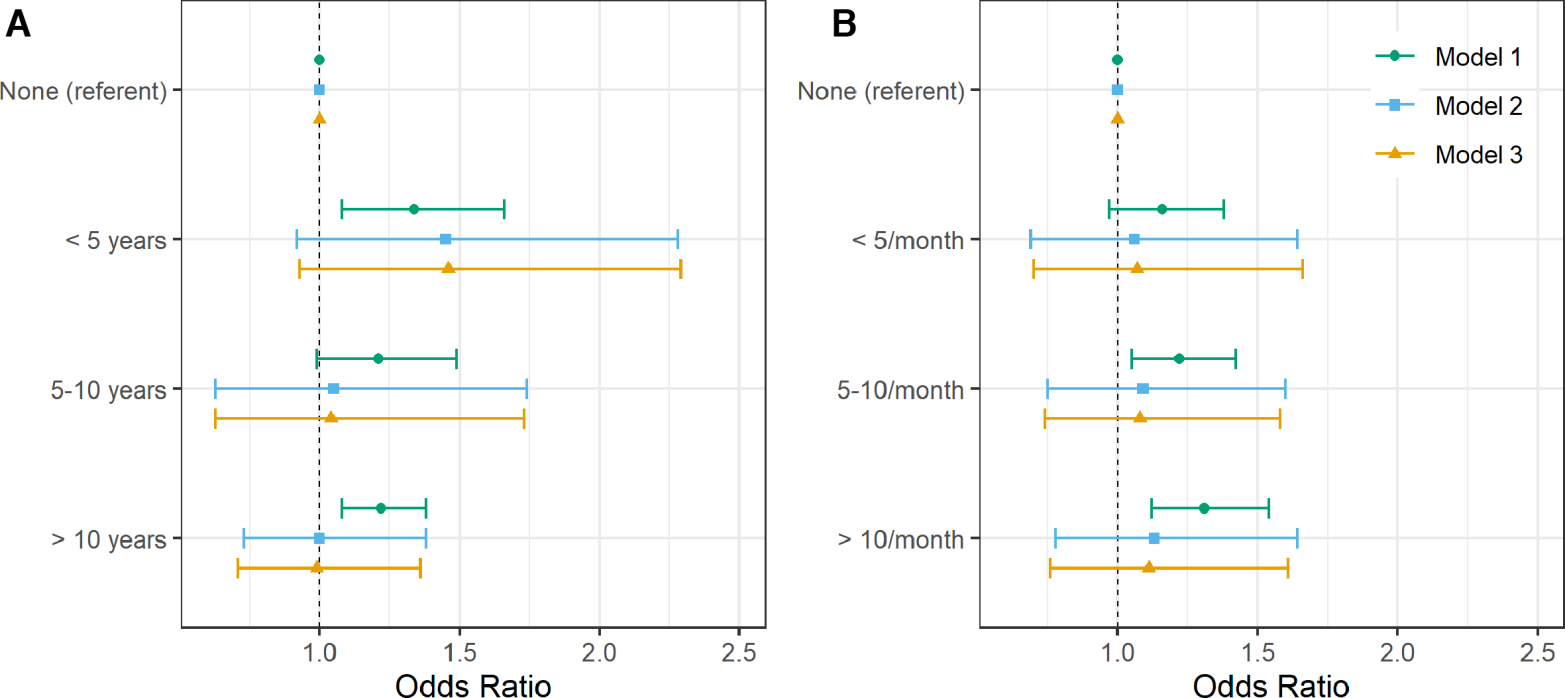
Adjusted odds of moderate-severe asthma by lifetime duration of shift work including nights (A) and by average monthly frequency of shifts that included night shifts (B) (n=107 930). Forest plot of adjusted ORs with corresponding 95% asymptotic CIs for moderate-severe asthma stratified by lifetime duration of shift work including nights (A) and by average monthly frequency of shifts that included nights (B). Three multivariate logistic regression models were fitted to the data: Model 1 (green circle): age- and sex-adjusted. Model 2 (blue square) covariates: age, sex, smoking status, smoking pack-years, alcohol status, daily alcohol intake, ethnicity, Townsend Deprivation Index, days exercised (walked, moderate and vigorous), body mass index, chronotype, length of working week, job asthma risk and job medical required. Model 3 (yellow triangle): model 2 covariates plus sleep duration.

Next we used data on 107 930 participants who provided lifetime work history data. When compared with those reporting no history of shift work, the highest odds for moderate-severe asthma was seen in participants reporting <5 years of shift work (OR 1.34 (95% CI 1.08 to 1.66)) and the lowest odds when performing ≥10 years of shift work (OR 1.22 (95% CI 1.08 to 1.38)) (figure 3A). In participants reporting <5 years of shift work, high point estimates for odds for moderate-severe asthma remained after adjusting for covariates in Models 2 and 3, but relationships were attenuated to the null for those with higher lifetime durations. No strong statistical evidence of a trend was found when treating lifetime duration of shift work as a continuous variable in any model.

### Average lifetime night shift frequency

Using the same historical lifetime work data, we analysed the prior frequency of night shift work in relation to the prevalence of moderate-severe asthma (n=107 930; figure 3B). In age- and sex-adjusted models, when compared with participants reporting no shift work, there were higher odds of moderate-severe asthma in people reporting prior higher frequencies of night shift work (5–10 night shifts/month): OR 1.22 (95% CI 1.05 to 1.42) and also ≥10 night shifts/month: OR 1.31 (95% CI 1.12 to 1.54), but not the lower frequency of shift work (<5 night shifts/month): OR 1.16 (95% CI 0.97 to 1.38). However, these associations attenuated to the null on covariate adjustment (Models 2 and 3). There was no strong statistical evidence of average lifetime frequency of night shift work as a continuous variable associating with asthma in any model.

### Chronotype

We analysed the likelihood of asthma by chronotype in all UK Biobank participants (n=413 040; table 3). People reporting either extreme chronotype (definitely a morning person or definitely an evening person) had higher odds of having any asthma compared with those describing themselves as intermediate chronotypes. After adjustment for covariates, the ORs for asthma in those reporting being definitely a morning person were 1.12 (95% CI 1.03 to 1.21) and 1.16 (95% CI 1.04 to 1.28) for those reporting being definitely an evening person (Model 3).

**Table 3:** Adjusted odds (95% CI) of any asthma by chronotype (n=413 040)

	Chronotype		
	Intermediate chronotype	Definitely a morning person	Definitely an evening person
Total cases (% of total sample size)	15 010 (5.68%)	6786 (6.06%)	2596 (7.06%)
Total sample size	264 279	112 007	36 754
Model 1: Age- and sex-adjusted OR (95% CI)	1 (referent)	1.07 (1.04 to 1.10)	1.27 (1.22 to 1.33)
Model 2: Multivariable adjusted OR (95% CI)	1 (referent)	1.12 (1.04 to 1.22)	1.16 (1.05 to 1.29)
Model 3: Model 2 covariates+sleep duration OR (95% CI)	1 (referent)	1.12 (1.03 to 1.21)	1.16 (1.04 to 1.28)

Model 2 covariates: age, sex, smoking status, smoking pack-years, alcohol status, daily alcohol intake, ethnicity, Townsend Deprivation Index, days exercised (walked, moderate and vigorous), body mass index, length of working week, job asthma risk and job medical required. Model 3 data are adjusted for Model 2 covariates plus sleep duration.

When we assessed the likelihood of moderate-severe asthma in relation to chronotype (n=398 252), age- and sex-adjusted models showed higher odds for moderate-severe asthma for people with either extreme chronotype compared with people with intermediate chronotypes (see online supplemental table 3). Results for definitely an evening person attenuated to the null after covariate adjustment (eg, Model 3: OR 1.17 (95% CI 0.99 to 1.38)); attenuation was less for definitely a morning person (eg, Model 3: OR 1.19 (95% CI 1.05 to 1.35)).

Finally, in relation to chronotype, we assessed the likelihood of moderate-severe asthma in individuals with a definite morning chronotype by shift work pattern (n=59 621; online supplemental table 4). In participants who reported being definitely a morning person there was a higher odds of moderate-severe asthma in covariate-adjusted models in those working irregular shifts, including nights, compared with those working day shifts (eg, Model 2: OR 1.55 (95% CI 1.06 to 2.27)). There was no excess risk for those morning chronotype workers either on permanent night shifts or rarely working nights.

There was no strong evidence of associations between shift work pattern and the likelihood of moderate-severe asthma when we restricted our analysis to individuals who reported being definitely an evening person (n=20 834) or being an intermediate chronotype (n=148 216) (see online supplemental table 4). There was no statistical evidence of an interaction between chronotype and shift work in association with asthma (p_interaction_=0.21).

### Asthma Genetic Risk Score (GRS)

We examined whether genetic susceptibility for asthma modified the relationship between shift work and the likelihood of asthma. In those of European ancestry in the UK Biobank cohort, we first showed that a higher genetic risk for asthma was associated with a higher odds of moderate-severe asthma (Model 2: per risk allele OR 1.13 (95% CI 1.11 to 1.16), p_trend_<0.01, n=313 816) and for risk of any asthma (Model 2: per risk allele OR 1.12 (95% CI 1.10 to 1.13), p_trend_<0.01, n=302 686). To investigate this effect further we split the GRS into quartiles and calculated odds of any asthma (online supplemental table 5) and moderate-severe asthma (online supplemental table 6) on these quartiles. Using the quartiles of GRS for moderate-severe asthma, we found that the asthma GRS had a statistically significant interaction on the relationships between odds for moderate-severe asthma and current shift work schedule (p<0.05). However, this interaction did not appear linear in its effects. Odds were higher for moderate-severe asthma in shift workers (who never or rarely worked night shifts) in the second GRS quartile (OR 1.78 (95% CI 1.17 to 2.68)) and also in permanent night shift workers in the third GRS quartile (OR 2.04 (95% CI 1.11 to 3.74), online supplemental table 7).

### Chronic obstructive pulmonary disease (COPD), emphysema and chronic bronchitis

Our definition of asthma may have included participants who had a concurrent doctor diagnosis of COPD, emphysema or chronic bronchitis since some medications are used to treat all conditions. There is no way of determining which condition is predominant among UK Biobank participants, therefore we re-analysed the cohort after excluding all cases of concurrent doctor-diagnosed COPD, emphysema and chronic bronchitis. A total of 1790 participants were removed from the any asthma group and 1572 participants from the moderate-severe asthma group. Our results were similar to our previous findings: for moderate-severe asthma we found, in an age- and sex-adjusted model, there was a higher odds of having moderate-severe asthma in day shift workers who never or rarely undertook night shifts (OR 1.12 (95% CI 1.01 to 1.24) when compared with day workers (see online supplemental table 8). After adjusting for additional covariates (Model 2), only permanent night shift workers had a significantly higher likelihood of asthma (OR 1.35 (95% CI 1.01 to 1.82)). Further adjustment for sleep duration slightly attenuated the likelihood of moderate-severe asthma in permanent night shift workers (OR 1.33 (95% CI 0.99 to 1.79)). In an age- and sex-adjusted model, we observed a higher likelihood of asthma in shift workers who never or rarely worked night shifts when compared with day workers (OR 1.07 (95% CI 1.01 to 1.14)). However, this association attenuated to the null after adjusting for additional covariates (Model 2). In contrast, the likelihood of asthma in shift workers working permanent nights was statistically significant in multivariable-adjusted models (Model 2: OR 1.26 (95% CI 1.05 to 1.50); Model 3: OR 1.23 (95% CI 1.03 to 1.48); see online supplemental table 9).

## Discussion

This study shows that, compared with day workers: (a) people working permanent nights had higher adjusted odds of moderate-severe asthma; (b) people doing any type of shift work had higher adjusted odds of wheeze or whistling in the chest; (c) shift workers who never or rarely worked on nights and people working permanent nights had a higher adjusted likelihood of having reduced lung function (FEV_1_ <80% predicted). We analysed data from more than 280 000 UK Biobank participants, 17% of whom were shift workers, which is similar to the reported prevalence of shift work in other industrialised countries.^7^

Rotational shift work disrupts the entrainment of endogenous circadian rhythms to external cues in the environment, resulting in circadian misalignment.^21^ Shift workers, especially those working night shifts, sleep at an inappropriate circadian phase, causing circadian misalignment between their sleep/wake behaviour and endogenous circadian processes. Mouse models of shift work have shown that it is the phase misalignment between the internal clockwork and behaviour that drives many of the resulting pathologies.^22^ To date, the association between asthma and circadian misalignment has not been investigated. We discover that night shift work associates with an increased risk of asthma.

As the UK Biobank data are drawn from a cross-sectional observational study, no causal inference is possible. However, it is plausible that circadian misalignment leads to asthma development. To investigate this we looked at people with extreme chronotypes (morning/evening preferences) who experience a degree of circadian misalignment in the absence of shift work exposure. We found that extreme chronotypes were significantly more likely to have asthma even after multivariable adjustments. The majority of individuals in our analysis were day workers (n=236 897), therefore the higher likelihood of asthma in evening types may represent circadian misalignment caused through conforming to early day shift working hours.^23^ In support of our findings, previous smaller studies have shown that evening or intermediate chronotypes associate with asthma.^24^ Our analysis of chronotype included data from 413 040 individuals including 9604 people with moderate-severe asthma. When we analysed chronotype in the context of type of shift work, we found that there was an increase in moderate/severe asthma risk in morning chronotypes working irregular shifts, including nights (OR 1.55 (95% CI 1.06 to 2.27)). Morning types find it particularly difficult to adjust to working night shifts^25^ and display the highest levels of circadian misalignment. Evening chronotypes showed no increase in the risk of asthma after shift work exposure, raising the intriguing possibility that evening chronotypes might be protected from the effects of shift work on asthma risk. Interestingly, chronotype does change with age, getting later through adolescence and then earlier as adults age,^26^ suggesting that older individuals might find it more difficult to adjust to night shift work than younger adults.

We found that the likelihood for any asthma and moderate-severe asthma was higher in individuals working permanent night shifts than in those working irregular shift work patterns, including nights. One might assume irregular night shifts lead to more circadian misalignment than permanent night shifts; however, only a small minority (<3%) of permanent night shift workers appear to adequately adjust their endogenous circadian timing to night work, as assessed by circadian rhythmicity of melatonin.^27^

We found a cumulative increase in the odds for moderate-severe asthma in shift workers working more frequent nights; however, this association was attenuated to the null after covariate adjustments and there was no strong statistical evidence when shift work frequency was treated as a continuous variable. We found higher odds of moderate-severe asthma in individuals who had worked night shifts for <5 years and in those who had worked for ≥10 years compared with day workers. The point estimates for the ORs suggested that this association might be stronger in individuals working night shifts for <5 years compared with those who had worked for ≥10 years. We postulate that this might represent the healthy worker effect, where individuals stop working night shifts once their health declines.^28^ However, these analyses need to be repeated in larger studies.

We devised a GRS for asthma derived from genome-wide association study (GWAS) signals^20^ and sought evidence that genetic susceptibility for asthma may modify the risk of shift work exposure. However, the emerging data were inconclusive, with associations being apparent in the middle two quarters of the GRS distribution and not consistent with stronger associations at higher genetic liability as we might have expected. Such an intersection between genetic risk of asthma and response to shift work exposure would require replication in a larger cohort.

Our study has several strengths; it involves a large cohort of >280 000 individuals from across the UK, with detailed medical history, current employment information, lifestyle information and demographic details, all of which were collected in a uniform manner. Of these >160 000 also had genetic data available. In addition, >100 000 individuals from the original cohort also provided a detailed employment history.

The study also has some limitations. First, UK Biobank participation rates were low at ~5%, which may have introduced selection bias towards more healthy individuals.^29^ In fact, the overall prevalence of asthma in all participants studied here was ~5% (also ~5% in the shift worker cohort alone) compared with ~10% within the general population of the UK.^30^ The UK Biobank provides no data on younger people and only limited data on ethnic minorities. The sample sizes were small for the morning and evening chronotype analyses, which resulted in low power. There was a reduction in sleep duration reported by night shift workers; this would be a potential confounder and so we took self-reported sleep duration into account in Model 3. In fact, we found that Model 3 did not significantly alter the results from Model 2. Furthermore, we determined differences in job type between day workers and shift workers and it is possible that these differences could account for some of our findings. To mitigate this risk, we took into account participants in jobs that might lead to occupational asthma or that required a medical assessment (selecting against asthma).

## Conclusion

This study shows that there is an increased likelihood of asthma (especially moderate-severe asthma) in shift workers on permanent nights. There are no specific national clinical guidelines for how to manage asthma in shift workers (or, indeed, when to measure lung function in night shift workers); however, modifying shift work schedules to take into account chronotype might present a public health measure to reduce the risk of developing inflammatory diseases such as asthma.
