# Supplementary figures and images for: Night shift work is associated with an increased risk of asthma
Source: Thorax. 2020 Nov 16;76(1):53–60. doi: 10.1136/thoraxjnl-2020-215218 (PMC7803886; doi:10.1136/thoraxjnl-2020-215218)

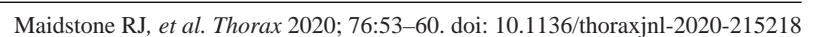

Supplement: Supplementary data [file thoraxjnl-2020-215218supp001.pdf]
