# Supplementary material for: Night shift work is associated with an increased risk of asthma
Source: Thorax. 2020 Nov 16;76(1):53–60. doi: 10.1136/thoraxjnl-2020-215218 (PMC7803886; doi:10.1136/thoraxjnl-2020-215218)
Supplement: Supplementary data [file thoraxjnl-2020-215218supp002.pdf]

Supplementary Methods

UK Biobank

The UK Biobank study was approved by the National Health Service National Research Ethics Service (ref. 11/NW/0382), and all participants provided written informed consent to participate in the UK Biobank study.

Shift Work Assessment

We defined shift work as previously reported by Vetter et al (1), however, we combined 'irregular or rotating shifts with some night shifts' and 'irregular or rotating shifts with ususal night shifts' to form one group 'irregular shift work including nights'. Briefly, participants employed at baseline were asked to report whether their current main job involved shift work (i.e. a schedule falling outside of 9:00am to 5:00pm; by definition, such schedules involved afternoon, eveninig or night shifts (or rotating though these shifts)). If yes, participants were further asked whether their main job involved night shifts, defined as '..a work schedule that involves working though the normal sleeping hours, for instance, working through the hours from 12:00am to 6:00am'. For both questions, response options were 'never/rarely', 'sometimes', 'usually', or 'always' and included additional options: 'prefer not to answer' and 'do not know'. Based on those two questions, we derived participants' current shift work status, categorized as 'day workers', 'shift worker, but only rarely if ever nights', 'irregular shift work including nights' and 'permanent night shifts'.

In the lifetime employment assessment, individuals reported each job ever worked, the number of years in each job ever worked, the number of years in each job, and the number of night shifts per month each job entailed. We restricted our analysis to those individuals

who provided in depth lifetime employment information (N= 107,930), we restricted the employment history to only jobs worked prior to 2008, since this was when the diagnosis of asthma was taken at baseline. We aggregated duration (i.e., number of years working night shifts) and frequency (i.e., the average number of night shifts per month) of night shift work.

Asthma Definition

Cases of asthma were defined by including all those participants with doctor diagnosed asthma at baseline as well as also being on any medication used to treat asthma as defined by Shrine et al. 2019 (2). Cases of moderate-severe asthma were defined as having doctor diagnosed asthma at baseline as well as meeting BTS step 3-5 criteria, i.e. for stage 3 taking β2 agonists plus inhaled corticosteroid; stage 4 taking higher dose inhaled corticosteroids than stage 3 patients and addition of a fourth drug (eg, leukotriene receptor antagonist, theophylline); and stage 5, taking oral corticosteroid or omalizumab, or both (2). We excluded participants with doctor-diagnosed asthma who reported not to be on asthma medication (N=18,806) and those on asthma medication but who did not have doctor diagnosed asthma (N=1,345) from our analyses. When analysing the risk of moderate-severe we further excluded participants with asthma taking medication on BTS stage 1 and 2 (N=9,455).

Within the parameters from the UK Biobank assessment centre data was the question relating to whether a participant had experienced 'Wheeze or whistling in the chest in the last year'. We excluded participants who answered "Do not know" or "Prefer not to answer" from any statistical analyses. Forced expiratory volume in 1-second (FEV_1), predicted percentage, was also analysed. FEV_1 predicted percentages were calculated (3). FEV_1 predicted percentages were filtered to produce two sub-populations; $FEV_1 \geq 80\%$ and FEV_1 <

80%, with the latter indicative of an obstructive respiratory pathology (4, 5) e.g. asthma (6, 7). Participants were split into ‘yes’ and ‘no’ sub-populations for ‘Wheeze or whistling in the chest in the last year’. These and the FEV_1 predicted percentage sub-populations were further split according to participant’s current work shift schedule, previously outlined.

Occupational Asthma

We identified participants who were employed in jobs that might lead to the development of occupational asthma. These jobs included bakers, food processors, forestry workers, chemical workers, plastics and rubber workers, metal workers, welders, textile workers, electrical and electronic production workers, storage workers, farm workers, waiters, cleaners, painters, dental workers and laboratory technicians (8-11). We also identified occupations, in which a medical assessment might select against a person with asthma (Protective Service Officers (officers in armed forces, police officers (inspectors and above) and senior officers in fire, ambulance, prison and related services), science technicians and researchers, probation officers and Transport Associate Professionals (including airline pilots and flight engineers, ship and hovercraft officers, train drivers). Both of these were included as covariates in models 2 and 3.

Chronotype

Participants self-reported chronotype on a touch-screen questionnaire at baseline by answering a question taken from the Morningness-Eveningness questionnaire (question 19;[12]). The question asks: “Do you consider yourself to be….” with response options “Definitely a ‘morning’ person”, “More a ‘morning’ than ‘evening’ person”, “More an ‘evening’ than a ‘morning’ person,” “Definitely an ‘evening’ person,” “Do not know,” and

"Prefer not to answer." Subjects who responded "Do not know" or "Prefer not to answer" were set to missing. This single item has been shown to correlate with sleep timing and dim-light melatonin onset (13-15). For our analyses we combined "more a 'morning' than 'evening' person" with "more an 'evening' than 'morning' person" to form an intermediate group. In our initial analysis of chronotype in asthma, we included all individuals with asthma and chronotype information, N= 413,040 (N=398,252 for moderate-severe asthma). Subsequently we investigated shift work in asthma stratified by chronotype (N = 228,671); this excluded participants not in paid employment or self-employed at baseline, or answered "Do not know" or "Prefer not to say" when asked (N=169,581).

Genetic Risk Score for Asthma

Genotyping in the UK Biobank was performed on two arrays, UK BiLEVE and UK Biobank Aziom. Genotyping, quality control, and imputation procedures have been previously described (16). A total of 488,232 participants in the UK Biobank were genotyped. In total, 337,409 unrelated samples of European ancestry were then filtered and those with an incomplete diagnosis of asthma were excluded, leaving 313,816 for analysis (302,686 for moderate/severe asthma).

We derived a genetic risk score (GRS) for asthma and moderate/severe asthma using 24 GWAS SNPs previously reported by Shrine et al. 2019 (2) for each individual participant. The GRS was generated using PLINK by summing the number of risk (asthma-increasing) alleles, which were weighted by the respective allelic effect size (β-coefficient) from the discovery GWAS. For variants not available in UK Biobank, we used the corresponding proxy SNP as indicated in Table 2 within (2). Scaling of the individual GRS was performed to allow interpretation of the effects as a per-1 risk allele increase in the GRS (division by twice the

sum of the β-coefficients and multiplication by twice the square of the SNP count representing the maximum number of risk alleles). Analysis of GRS was performed by subdividing into quartiles, as well as the impact per-1 risk allele. Analysis of the shift work effect on asthma was performed on all GRS quartiles. The interaction between GRS quartiles and shift work schedule was tested and a P value for interaction was computed.

When investigating continuous variables (lifetime duration and frequency of shift work including nights (**Figure 3**), and odds by genetic risk score (**Supplementary Tables 6 and 7**) p-values for the linear trend were obtained by considering the variable as continuous and running a Wald test to calculate the significance of the variable in our models.

To analyse the effect of GRS and chronotype on the relationship of current job shift schedule on asthma risk we compared models with and without an interaction term (between job shift schedule and GRS/chronotype). The two models were compared using a likelihood ratio test and a p-value indicating the significance of the interaction computed.

Supplemental References

1. Vetter C, Dashti HS, Lane JM, et al. Night Shift Work, Genetic Risk, and Type 2 Diabetes in the UK Biobank. Diabetes Care. 2018;41(4):762–769. doi:10.2337/dc17-1933
2. Shrine N, Portelli MA, John C, Soler Artigas M, Bennett N, Hall R, Lewis J, Henry AP, Billington CK, Ahmad A, Packer RJ, Shaw D, Pogson ZEK, Fogarty A, McKeever TM, Singapuri A, Heaney LG, Mansur AH, Chaudhuri R, Thomson NC, Holloway JW, Lockett GA, Howarth PH, Djukanovic R, Hankinson J, Niven R, Simpson A, Chung KF, Sterk PJ, Blakey JD, Adcock IM, Hu S, Guo Y, Obeidat M, Sin DD, van den Berge M,

Nickle DC, Bossé Y, Tobin MD, Hall IP, Brightling CE, Wain LV, Sayers I. Moderate-to-severe asthma in individuals of European ancestry: a genome-wide association study. Lancet Respir Med. 2019 Jan;7(1):20-34. doi: 10.1016/S2213-2600(18)30389-8. Epub 2018 Dec 11.

3. Wain LV, Shrine N, Miller S et al. Novel insights into the genetics of smoking behaviour, lung function, and chronic obstructive pulmonary disease (UK BiLEVE): a genetic association study in UK Biobank. Lancet Respir Med. 2015; 3: 769-781
4. Gauderman, W. J., Avol, E., Gilliland, F., Vora, H., Thomas, D., Berhane, K., McConnell, R., Kuenzli, N., Lurmann, F. & Rappaport, E. (2004). 'The effect of air pollution on lung development from 10 to 18 years of age', New England Journal of Medicine, 351(11), pp. 1057-1067.
5. Criner, G. J., Martinez, F. J., Anzueto, A., Barnes, P. J. & Bourbeau, J. (2017). 'Global Strategy for the Diagnosis, Management and Prevention of Chronic Obstructive Lung Disease 2017 Report'.
6. Global Initiative for Asthma, P. (2017). 'Global Strategy for Asthma Management and Prevention: 2017 Update'.
7. Cukic, V., Lovre, V., Dragisic, D. & Ustamujic, A. (2012). 'Asthma and chronic obstructive pulmonary disease (COPD)–differences and similarities', Materia socio-medica, 24(2), p. 100.46. BTS/SIGN British Guideline on the management of asthma 2019
8. Jaakkola JJ, Pilpari R, Jaakkola MS. Occupation and asthma; a population-based incident case-control study. Am J Epidemiol 2003;158:981-7

9. Johnson AR, Dimich-Ward HD, Manfreda J, Becklake MR, Ernst P, Sears MR, Bowie DM, Sweet L, Chan-Yeung M. Occupational asthma in adults in six Canadian communities. Am J Respir Crit Care Med 2000;162:2058-62
10. Kogevinas M, Anto JM, Soriano JB, Tobias A, Burney P. The risk of asthma attributable to occupational exposures. A population-based study in Spain. Spanish Group of the European Asthma Study. Am J Respir Crit Care Med 1996;154:137-43
11. Kogevinas M, Anto JM, Sunyer J, Tobias A, Kromhout H, Burney P. Occupational asthma in Europe and other industrialised areas: a population-based study. European Community Respiratory Health Survey Study Group. Lancet 1999;353:1750-4
12. Horne JA, Ostberg O. A self-assessment questionnaire to determine morningness-eveningness in human circadian rhythms. Int J Chronobiol 1976;4:97-110
13. Kitamura S, Hilda A, Aritake S et al. Validity of the Japanese version of the Munich Chronotype Questionnaire. Chronobiol INt 2014;31:845-850.
14. Megdal SP, Schernhammer ES. Correlates for poor sleepers in a Los Angeles high school. Sleep Med 2007;9:60-63
15. Taillard J, Philip P, Chastang JF, Bioulac B. Validation of Horne and Ostberg morningness eveningness questionnaire in a middle-aged population of French workers. J Biol Rythms 2004;19:76-86
16. Lane JM, Vlasac I, Anderson SG, et al. Genome-wide association analysis identifies novel loci for chronotype in 100,420 individuals from the UK Biobank. Nat Commun 2016;7:10889

Supplementary Figure 1: STROBE diagram showing filtering of participants for each analysis. STROBE diagram showing how the full UK Biobank cohort (N=502,540) was filtered for each analysis. Blue boxes correspond to individuals used for the analyses resulting in each figure/table. White boxes show excluded participants at each stage.

Supplementary Table 1: Job Standard Occupational Classification (SOC) category splits by current night shift work exposure (N = 76,162)

	Current work schedule			
	Day workers	Shift work, but never or rarely night shifts	Irregular shift work including nights	Permanent night shift work
N	66360	4986	3640	1176
Time in most recent job (years)	20.46 (13.01)	20.25 (13.41)	21.62 (13.26)	23.77 (13.34)
Managers and Senior Officials (%)	17.49	12.8	9.59	6.21
Professional Occupations (%)	36.29	18.47	14.67	5.7
Associate Professional and Technical Occupations (%)	18.25	29.1	39.67	38.27
Administrative and Secretarial Occupations (%)	14.27	9.73	4.48	6.55
Skilled Trades Occupations (%)	4.12	6.28	8.74	7.4
Personal Service Occupations (%)	3.64	7.82	8.05	10.03
Sales and Customer Service Occupations (%)	2.39	4.83	2.01	1.87
Process, Plant and Machine Operatives (%)	1.54	6.14	9.09	13.69
Elementary Occupations (%)	2.03	4.83	3.71	10.29

Data are mean (SD) or percentages.

BMJ Publishing Group Limited (BMJ) disclaims all liability and responsibility arising from any reliance placed on this supplemental material which has been supplied by the author(s)

Supplementary Table 2: Adjusted odds (95% CI) of any asthma by current shift work exposure (N=266,674)

	Current work schedule			
	Day workers	Shift work, but never or rarely night shifts	Irregular shift work including nights	Permanent night shift work
Total cases (% of total sample size)	11,695 (5.31%)	1,306 (5.72%)	872 (5.15%)	365 (5.48%)
Total sample size	220,234	22,838	16,945	6,657
Model 1: Age and Sex adjusted OR (95% CI)	1 (referent)	1.08 (1.02-1.15)	0.98 (0.91-1.05)	1.05 (0.95-1.17)
Model 2: Multivariable adjusted OR (95% CI)	1 (referent)	1.06 (0.95-1.18)	1.08 (0.95-1.22)	1.23 (1.03-1.46)
Model 3: Model 2 covariates + Sleep Duration (95% CI)	1 (referent)	1.06 (0.95-1.18)	1.07 (0.94-1.21)	1.20 (1.01-1.43)

Model 2 covariates: age, sex, smoking status, smoking pack years, alcohol status, daily alcohol intake, ethnicity, Townsend deprivation index, days exercised (walked, moderate and vigorous), BMI, chronotype, length of working week, job asthma risk and job medical required. Model 3 data are adjusted for Model 2 covariates plus sleep duration.

BMJ Publishing Group Limited (BMJ) disclaims all liability and responsibility arising from any reliance placed on this supplemental material which has been supplied by the author(s)

Supplementary Table 3: Adjusted odds (95% CI) of moderate-severe asthma by chronotype (N = 398,252)

	Chronotype		
	Intermediate chronotype	Definitely a morning person	Definitely an evening person
Total cases (% of total sample size)	5,820 (2.28%)	2,782 (2.58%)	1,002 (2.85%)
Total sample size	255,089	108,003	35,160
Model 1: Age and Sex adjusted OR (95% CI)	1 (referent)	1.10 (1.06-1.16)	1.30 (1.21-1.39)
Model 2: Multivariable adjusted OR (95% CI)	1 (referent)	1.19 (1.05-1.36)	1.18 (0.99-1.39)
Model 3: Model 2 covariates + Sleep Duration (95% CI)	1 (referent)	1.19 (1.05-1.35)	1.17 (0.99-1.38)

Model 2 covariates: age, sex, smoking status, smoking pack years, alcohol status, daily alcohol intake, ethnicity, Townsend deprivation index, days exercised (walked, moderate and vigorous), BMI, length of working week, job asthma risk and job medical required. Model 3 data are adjusted for Model 2 covariates plus sleep duration.

Supplementary Table 4: Adjusted odds (95% CI) and association of moderate-severe asthma and current shift work exposure by chronotype

Current work schedule	OR (95% CI)	$P_{interaction}$
**Definite morning chronotype (N= 59,621, 1,216 cases)**		
Day workers	1 (referent)	0.21
Shift work, but never or rarely night shifts	0.97 (0.67-1.39)	
Irregular shift work including nights	1.55 (1.06-2.27)	
Permanent night shift work	1.32 (0.69-2.51)	
**Intermediate chronotype (N= 148,216, 2,645 cases)**		
Day workers	1 (referent)	
Shift work, but never or rarely night shifts	1.13 (0.90-1.43)	
Irregular shift work including nights	1.11 (0.84-1.47)	
Permanent night shift work	1.33 (0.88-2.00)	
**Definite evening chronotype (N= 20,834, 447 cases)**		
Day workers	1 (referent)	
Shift work, but never or rarely night shifts	1.18 (0.70-1.99)	
Irregular shift work including nights	1.10 (0.61-1.99)	
Permanent night shift work	1.52 (0.88-2.65)	

Models were adjusted for covariates in model 2 (age, sex, smoking status, smoking pack years, alcohol status, daily alcohol intake, ethnicity, Townsend deprivation index, days exercised (walked, moderate and vigorous), BMI, length of working week, job asthma risk and job medical required).
Interaction p-value is calculated using a LR test comparing the model with and without an interaction term.

Supplementary Table 5: Adjusted odds (95% CI) of moderate-severe asthma by genetic risk score (GRS) quartile (N = 302,686)

	GRS quartile				p-value for trend
	1st quartile	2nd quartile	3rd quartile	4th quartile	
Total cases (% of total sample size)	1,166 (1.50%)	1,585 (2.07%)	1,906 (2.53%)	2,707 (3.71%)	
Total sample size	77,746	76,580	75,435	72,925	
Model 1: Age and Sex adjusted OR (95% CI)	1 (referent)	1.39 (1.29-1.50)	1.70 (1.58-1.83)	2.53 (2.36-2.71)	<0.01
Model 2: Multivariable adjusted OR (95% CI)	1 (referent)	1.22 (0.99-1.52)	1.70 (1.39-2.08)	2.66 (2.21-3.22)	<0.01
Model 3: Model 2 covariates + Sleep Duration (95% CI)	1 (referent)	1.23 (0.99-1.52)	1.71 (1.40-2.09)	2.67 (2.21-3.23)	<0.01

Model 2 covariates: age, sex, smoking status, smoking pack years, alcohol status, daily alcohol intake, Townsend deprivation index, days exercised (walked, moderate and vigorous), BMI, chronotype, length of working week, job asthma risk and job medical required. Model 3 data are adjusted for Model 2 covariates plus sleep duration.

Supplementary Table 6: Adjusted odds (95% CI) of any asthma by genetic risk score (GRS) quartile (N = 313,816)

	GRS quartile				p-value for trend
	1st quartile	2nd quartile	3rd quartile	4th quartile	
Total cases (% of total sample size)	3,106 (3.90%)	3,942 (4.99%)	4,818 (6.15%)	6,628 (8.63%)	
Total sample size	79,686	78,937	78,347	76,846	
Model 1: Age and Sex adjusted OR	1 (referent)	1.30 (1.23-1.36)	1.62 (1.54-1.69)	2.33 (2.23-2.43)	<0.01
Model 2: Multivariable adjusted OR (95% CI)	1 (referent)	1.24 (1.09-1.41)	1.52 (1.35-1.72)	2.33 (2.08-2.61)	<0.01
Model 3: Model 2 covariates + Sleep Duration (95% CI)	1 (referent)	1.24 (1.10-1.41)	1.53 (1.35-1.72)	2.33 (2.08-2.61)	<0.01

Model 2 covariates: age, sex, smoking status, smoking pack years, alcohol status, daily alcohol intake, Townsend deprivation index, days exercised (walked, moderate and vigorous), BMI, chronotype, length of working week, job asthma risk and job medical required. Model 3 data are adjusted for Model 2 covariates plus sleep duration.

Supplementary Table 7: Adjusted odds (95% CI) and association of moderate-severe asthma and current shift work exposure by genetic risk

Current work schedule	OR (95% CI)	$P_{interaction}$
**GRS first quartile (lowest) (N= 44,088, 475 cases)**		
Day workers	1 (referent)	<0.05
Shift work, but never or rarely night shifts	1.18 (0.71-1.96)	
Irregular shift work including nights	0.74 (0.35-1.53)	
Permanent night shift work	1.67 (0.80-3.51)	
**GRS second quartile (N= 43,396, 667 cases)**		
Day workers	1 (referent)	
Shift work, but never or rarely night shifts	1.78 (1.17-2.68)	
Irregular shift work including nights	1.26 (0.72-2.23)	
Permanent night shift work	1.57 (0.75-3.27)	
**GRS third quartile (N= 42,507, 846 cases)**		
Day workers	1 (referent)	
Shift work, but never or rarely night shifts	1.41 (0.95-2.09)	
Irregular shift work including nights	1.22 (0.74-2.02)	
Permanent night shift work	2.04 (1.11-3.74)	
**GRS fourth quartile (highest) (N= 40,905, 1,218 cases)**		
Day workers	1 (referent)	
Shift work, but never or rarely night shifts	0.93 (0.64-1.35)	
Irregular shift work including nights	1.42 (0.97-2.10)	
Permanent night shift work	1.52 (0.90-2.56)	

Models were adjusted for covariates in model 2 (age, sex, smoking status, smoking pack years, alcohol status, daily alcohol intake, ethnicity, Townsend deprivation index, days exercised (walked, moderate and vigorous), BMI, chronotype, length of working week, job asthma risk and job medical required). Interaction p-value is calculated using a LR test comparing the model with and without an interaction term.

Supplementary Table 8: Adjusted odds (95% CI) of moderate-severe asthma by current shift work exposure after excluding participants with doctor diagnosed Chronic Obstructive Pulmonary Disease (COPD), emphysema or chronic bronchitis (N = 255,647)

	Current work schedule			
	Day workers	Shift work, but never or rarely night shifts	Irregular shift work including nights	Permanent night shift work
Total cases (% of total sample size)	3,668 (1.74%)	418 (1.92%)	267 (1.65%)	119 (1.87%)
Total sample size	211,283	21,787	16,225	6,352
Model 1: Age and Sex adjusted OR (95% CI)	1 (referent)	1.12 (1.01-1.24)	1.02 (0.90-1.15)	1.15 (0.96-1.39)
Model 2: Multivariable adjusted OR (95% CI)	1 (referent)	1.15 (0.96-1.39)	1.13 (0.90-1.40)	1.35 (1.01-1.82)
Model 3: Model 2 covariates + Sleep Duration (95% CI)	1 (referent)	1.15 (0.96-1.38)	1.12 (0.90-1.39)	1.33 (0.99-1.79)

Model 2 covariates: age, sex, smoking status, smoking pack years, alcohol status, daily alcohol intake, ethnicity, Townsend deprivation index, days exercised (walked, moderate and vigorous), BMI, chronotype, length of working week, job asthma risk and job medical required. Model 3 data are adjusted for Model 2 covariates plus sleep duration.

Supplementary Table 9: Adjusted odds (95% CI) of any asthma by current shift work exposure after excluding participants with doctor diagnosed Chronic Obstructive Pulmonary Disease (COPD), emphysema or chronic bronchitis (N = 264,884)

	Current work schedule			
	Day workers	Shift work, but never or rarely night shifts	Irregular shift work including nights	Permanent night shift work
Total cases (% of total sample size)	11,290 (5.16%)	1,247 (5.51%)	823 (4.90%)	349 (5.30%)
Total sample size	218,905	22,616	16,781	6,582
Model 1: Age and Sex adjusted OR (95% CI)	1 (referent)	1.07 (1.01-1.14)	0.96 (0.89-1.03)	1.04 (0.93-1.16)
Model 2: Multivariable adjusted OR (95% CI)	1 (referent)	1.04 (0.93-1.16)	1.05 (0.92-1.19)	1.26 (1.05-1.50)
Model 3: Model 2 covariates + Sleep Duration (95% CI)	1 (referent)	1.04 (0.93-1.16)	1.04 (0.91-1.18)	1.23 (1.03-1.48)

Model 2 covariates: age, sex, smoking status, smoking pack years, alcohol status, daily alcohol intake, ethnicity, Townsend deprivation index, days exercised (walked, moderate and vigorous), BMI, chronotype, length of working week, job asthma risk and job medical required. Model 3 data are adjusted for Model 2 covariates plus sleep duration.
